# Two cases of pineal-region meningiomas derived from arachnoid membrane over the vein of Galen without dural attachment

**DOI:** 10.1186/s12957-015-0645-z

**Published:** 2015-07-25

**Authors:** Akihiro Inoue, Takanori Ohnishi, Shohei Kohno, Yoshihiro Ohtsuka, Yawara Nakamura, Yosuke Mizuno, Riko Kitazawa, Shiro Ohue

**Affiliations:** Department of Neurosurgery, Ehime University School of Medicine, 454 Shitsukawa, Toon, Ehime 791-0295 Japan; Division of Diagnostic Pathology, Ehime University Hospital, 454 Shitsukawa, Toon, Ehime 791-0295 Japan

**Keywords:** Pineal-region meningioma, Arachnoid membrane over the vein of Galen, Occipital transtentorial approach, New entity of meningioma

## Abstract

**Background:**

We present two rare cases of pineal-region meningiomas. These tumors are the first reported cases of dura-unrelated meningiomas originating from the arachnoid membrane over the vein of Galen (AMG).

**Case description:**

In Case 1, a 37-year-old woman presented with a progressing headache. Magnetic resonance imaging (MRI) showed a large tumor in the pineal region, displacing the vein of Galen upward. Angiography disclosed occlusion of the vein of Galen, with deep venous flow draining through the veins on the right medial surface of the occipital lobe to the superior sagittal sinus. In Case 2, a 67-year-old man presented with dizziness. MRI demonstrated a large mass in the pineal region, displacing the vein of Galen inferiorly. Angiography disclosed occlusion of the vein of Galen, with deep venous flow draining through the collateral venous channel into the transverse sinus. Both tumors were totally excised (Simpson Grade III for Case 1, Grade I for Case 2) via a left occipital transtentorial approach. No dural attachment was recognized in either case, but the tumor in Case 1 was firmly adherent to the inferior portion of the AMG, while that in Case 2 was attached to the superior portion of the AMG, but remained dissectible.

**Conclusions:**

We reported two cases of pineal-region meningiomas originating from the arachnoid membrane over the vein of Galen, resulting in meningioma without dural attachment. These tumors can be totally resected by careful dissection of the tumor from the arachnoid membrane surrounding the vein of Galen.

## Background

Pineal-region meningiomas are uncommon, accounting for 2–8 % of all tumors in this area [1–8]. These tumors are characterized by meningioma occupying the quadrigeminal cistern and showing little or no dural attachment. To date, two types of such meningioma have been reported [1–8], occurring at the falcotentorial junction or velum interpositum. The tumor origin of velum interpositum meningioma is thought to be arachnoid cap cells within the outer arachnoid membrane derived from the arachnoid envelope over the pineal region (AEPR) [4, 5, 7–9]. On the other hand, the origin of falcotentorial meningioma has not been clearly defined. We recently encountered two cases of pineal-region meningioma that originated from the arachnoid membrane over the vein of Galen. We present the clinical features of these cases and discuss the significance of the microsurgical anatomy for safe and effective resection of this type of meningioma.

## Case presentation

### Case 1

**Fig. 1:**
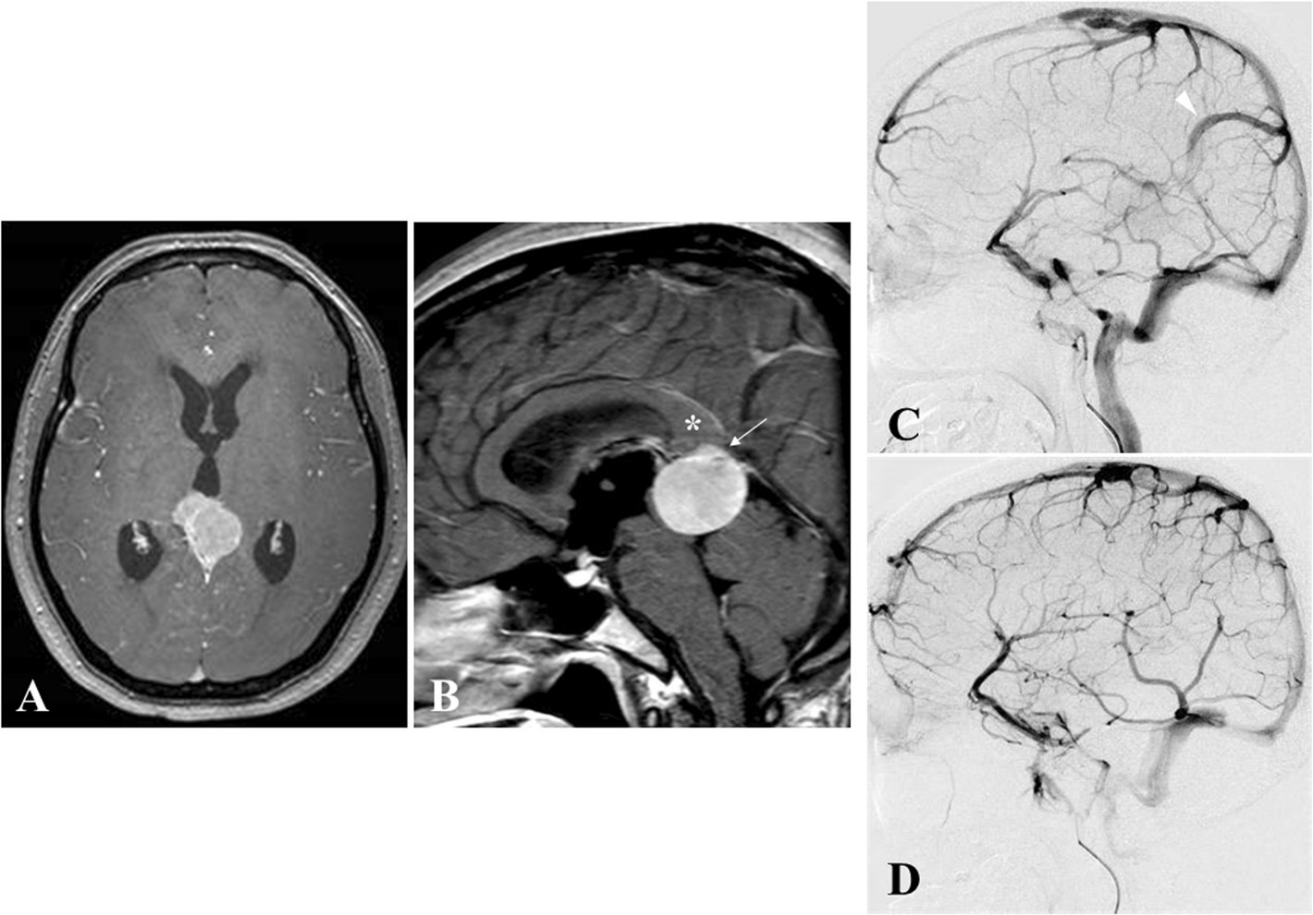
Radiological imaging of the tumor in the pineal region in Case 1. **a**, **b** Gadolinium-enhanced T1-weighted magnetic resonance imaging (MRI) in Case 1 shows a well-defined tumor within the quadrigeminal cistern, compressing the dorsal part of the midbrain with resulting mild hydrocephalus (**a**, axial view, **b** sagittal view). The vein of Galen (*white arrow*) and splenium of the corpus callosum (*white asterisk*) are displaced upward and the pineal body cannot be identified. **c**, **d** Preoperative internal cerebral angiography (ICAG) shows occlusion of the vein of Galen and development of a large collateral venous channel (*white arrowhead*) on the right medial surface of the occipital lobe, draining into the superior sagittal sinus (**c**, right venous phase, **d** left venous phase)

**Fig. 2:**
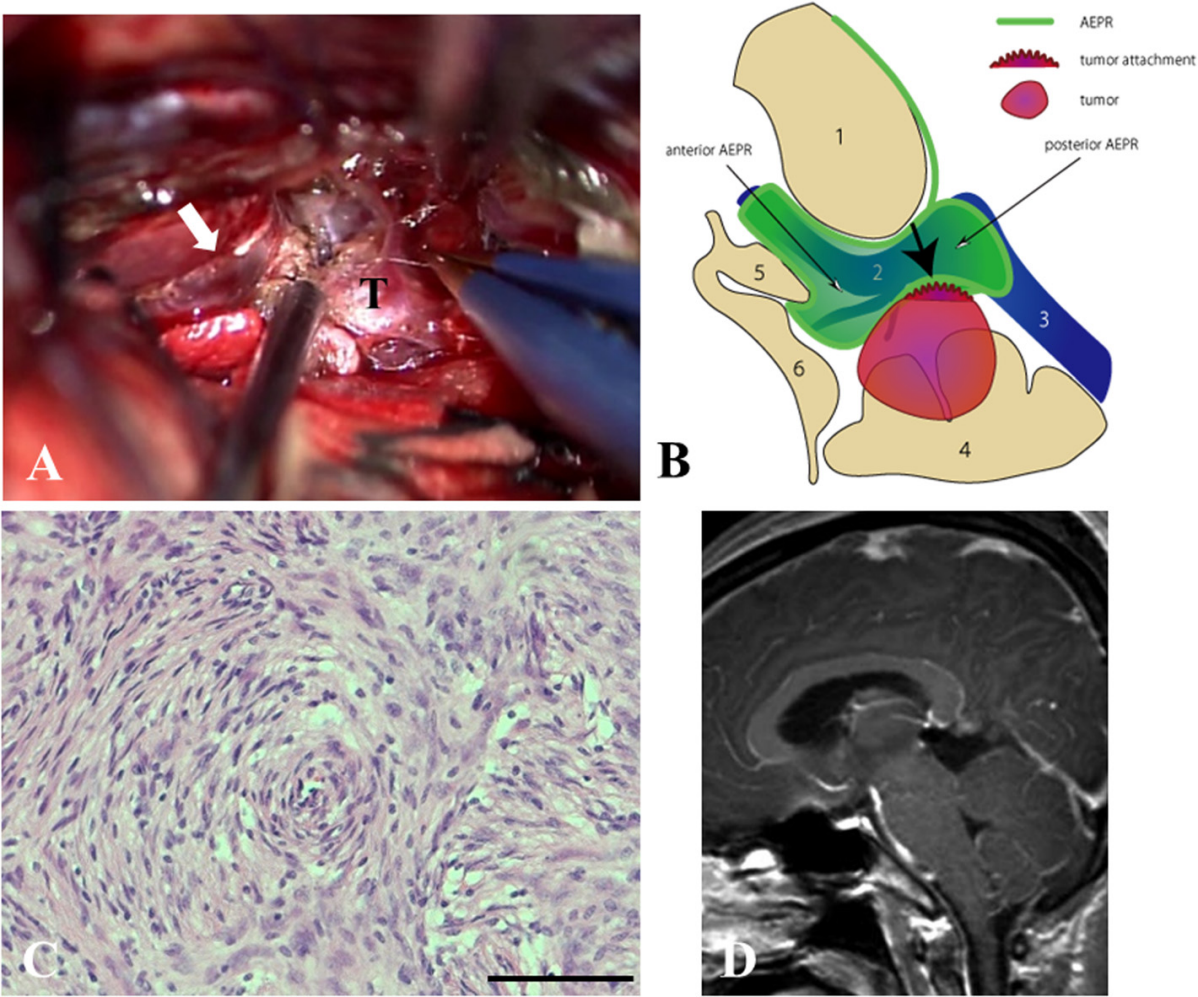
Intraoperative view and its illustration, histological examination, and postoperative MR imaging in Case1. **a** Surgical view shows a tumor tightly adherent to the inferior part of the vein of Galen (*white arrow*) *T* **,** tumor. **b** Intraoperative illustration of the microanatomical relationship between the tumor origin and arachnoid membranes from the perspective of the lower side of the vein of Galen (*black bold arrow* tumor attachment, *1* splenium, *2* vein of Galen, *3* straight sinus, *4* cerebellum, *5* pineal gland, *6* quadrigeminal plate). **c** Histological examination shows transitional meningioma (hematoxylin and eosin stain, scale bar, 100 μm). **d** MRI at 1 month postoperatively shows gross total removal of the tumor mass

A 37-year-old woman visited our department after experiencing a gradually worsening headache over a 6-month period. On admission, she presented with headache and altered mental status. Brain computed tomography (CT) and gadolinium (Gd)-enhanced magnetic resonance imaging (MRI) showed a homogeneously enhanced and well-defined tumor (diameter, 30 × 30 × 25 mm) in the pineal region. The tumor was located in the inferior portion of the vein of Galen, compressing the vessel upward. The splenium of the corpus callosum was also displaced superiorly **(**Fig. 1). Bilateral internal cerebral angiography (ICAG) revealed that apparent arterial supply was not visualized. The vein of Galen was occluded, and an upward shift of bilateral internal cerebral veins (ICVs) was not confirmed. Collateral venous flow drained through veins on the right medial surface of the occipital lobe to the superior sagittal sinus. Tumor resection was performed using a left occipital transtentorial approach (OTA). When the cerebellar tentorium was incised along the straight sinus to the free edge, a very thick and cloudy arachnoid membrane came into view, making it difficult to dissect the tumor from the vein of Galen and its tributaries. No dural attachment was seen at the falcotentorial junction or free edge of the tentorium. The tumor was firmly adherent to the inferior portion of the vein of Galen (Fig. 2a, b). The tumor was easily dissected from surrounding structures, including the brainstem, but we could not free the tumor from the attachment site that firmly adhered to the inferior thick wall surrounding the vein of Galen, resulting in a “Simpson Grade III” resection. Histological examination showed transitional meningioma, and Ki-67 (MIB-1) labeling index was low (2.6 %) (Fig. 2c). The headache was improved immediately after surgery, and no other abnormalities were identified. MRI at 3 months postoperatively did not show any tumor (Fig. 2d), and mental activity returned to near-normal. As of the time of writing, 1 year after surgery, the patient remains healthy without tumor recurrence.

### Case 2

**Fig. 3:**
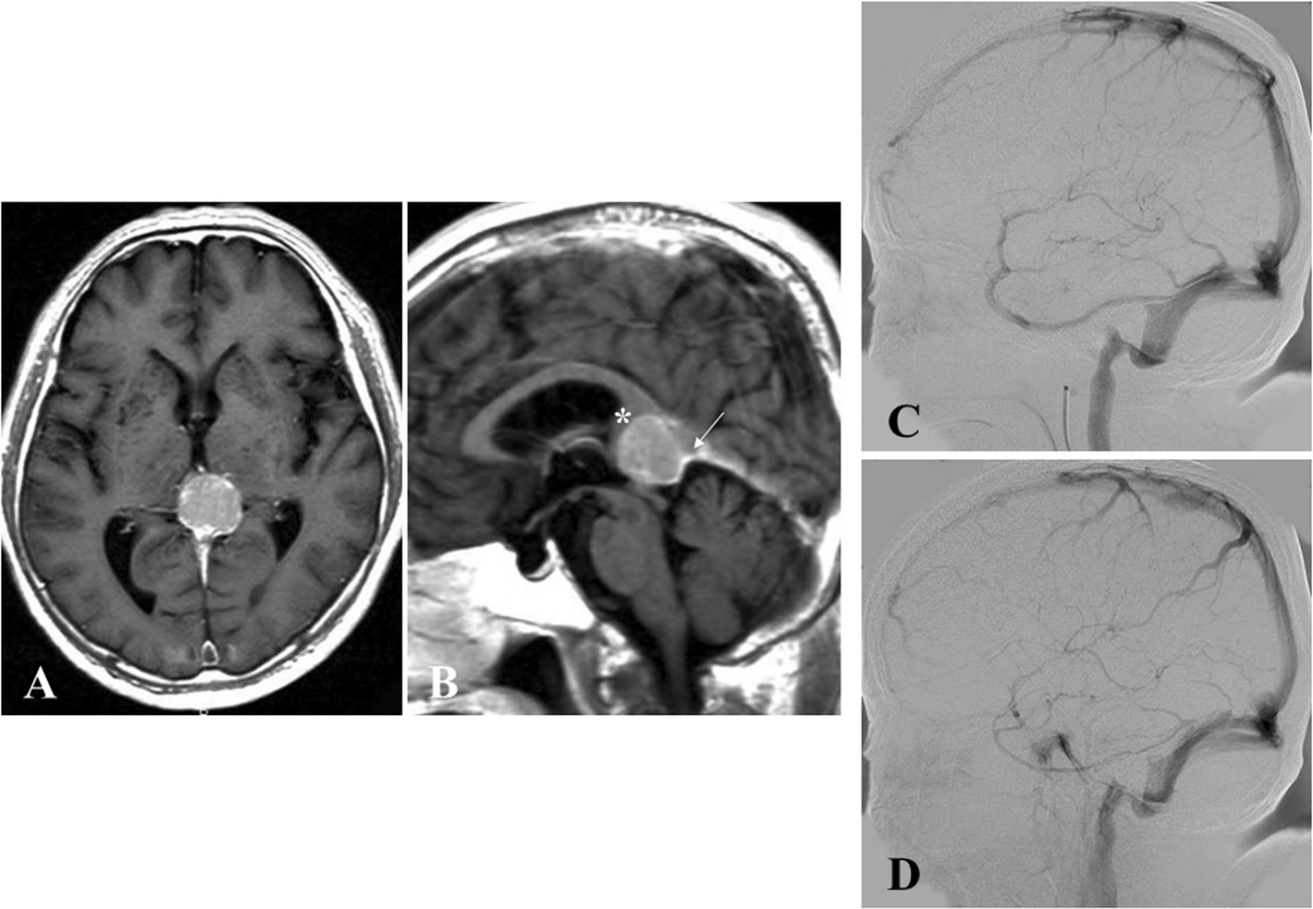
Radiological imaging of the tumor in the pineal region in Case 2. **a**, **b** Gadolinium-enhanced T1-weighted imaging in Case 2 shows a well-demarcated tumor in the quadrigeminal cistern, compressing the pineal gland and splenium of the corpus callosum (*white asterisk*) anteriorly (**a**, axial view, **b** sagittal view). The vein of Galen (*white arrow*) is displaced downward. **c, d** Preoperative ICAG shows occlusion of the vein of Galen and deep venous flow draining through the collateral venous channel into the transverse sinus (**c** right venous phase, **d** left venous phase)

**Fig. 4:**
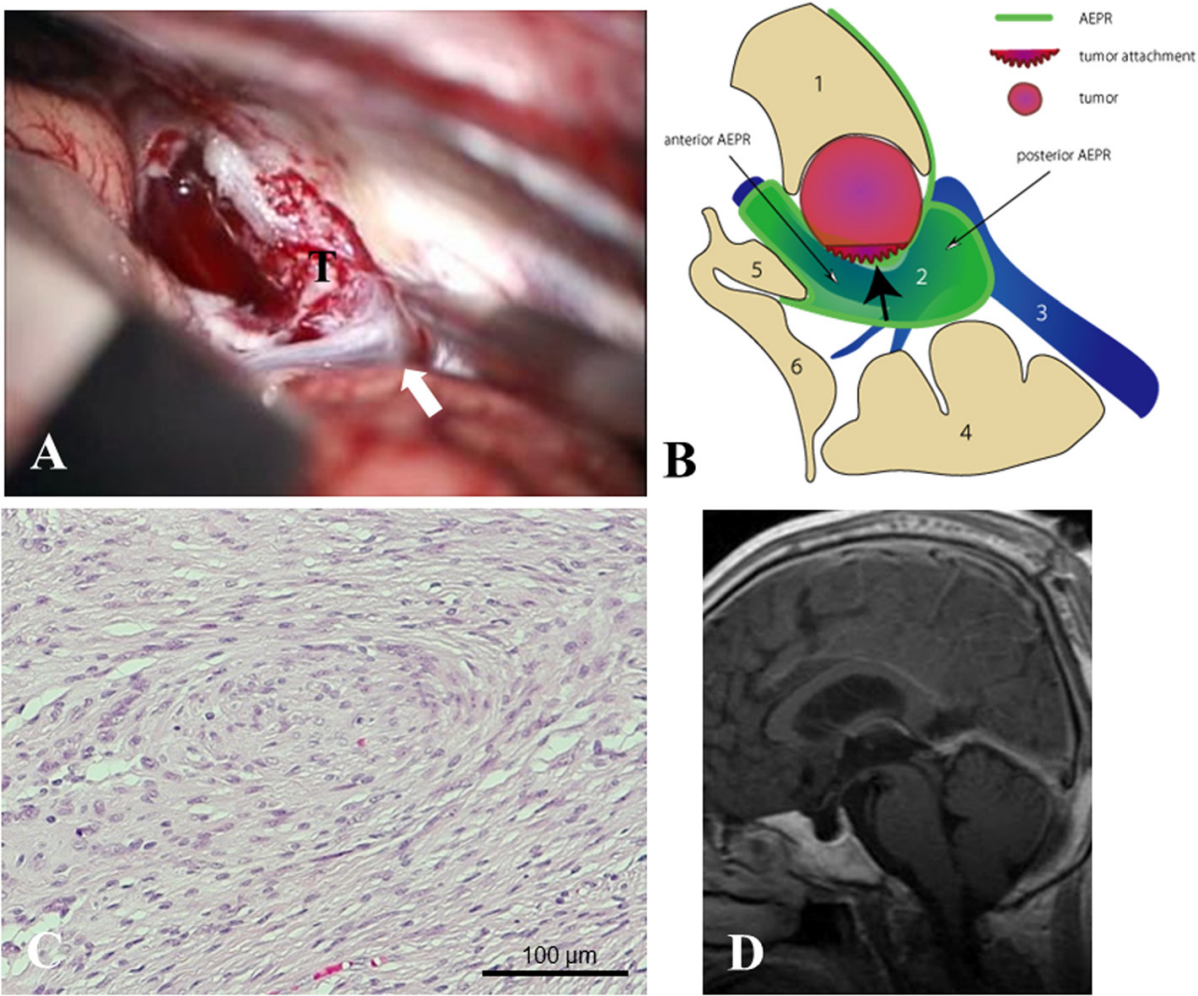
Intraoperative view and its illustration, histological examination and postoperative MR imaging in Case 2. **a** Surgical view shows a tumor attached to the superior part of the vein of Galen (white arrow). *T* tumor. **b** Intraoperative illustration of the microanatomical relationship between the tumor origin and arachnoid membranes from the perspective of the upper side of the vein of Galen (*black bold arrow* tumor attachment, *1* splenium, *2* vein of Galen, *3* straight sinus, *4* cerebellum, *5* pineal gland, *6* quadrigeminal plate). **c** Histological examination shows fibrous meningioma (hematoxylin and eosin stain, scale bar, 100 μm). **d** MRI at 1 month postoperatively shows gross total removal of the tumor mass

A 67-year-old man presented with a 6-month history of gradually progressing dizziness. No neurological deficits were seen at the time of admission. Gd-enhanced MRI demonstrated a large mass (30 mm in maximal diameter) in the pineal region. The tumor was located at the superior portion of the vein of Galen and compressed the splenium of the corpus callosum anteriorly. In addition, the tumor extended slightly to the left side (Fig. 3a, b). On angiography, the vein of Galen was occluded, and deep venous flows drained through the collateral venous channel into the transverse sinus (Fig. 3c, d). No upward shift of bilateral ICVs was demonstrated. The tumor was resected via a left OTA. When incising the tentorium to the free edge, the arachnoid membrane in front of the tumor was slightly cloudy, but not particularly tough, and was easily dissected to expose the tumor. No dural attachment of the tumor was observed at either the falcotentorial junction or the free edge of the tentorium. The tumor was adherent to the superior part of the vein of Galen and was able to be dissected from the vein relatively easily (Fig. 4a, b). The tumor was able to be totally resected (Simpson Grade I), and no postoperative complications were encountered. Histological examination showed fibrous meningioma (Fig. 4c). MRI at 3 months after surgery did not show any abnormalities (Fig. 4d). As of the time of writing, 6 years postoperatively, the patient remains healthy and without tumor recurrence.

The clinical study of the above-mentioned case reports was approved by the Ethics Committee for Clinical Research of Ehime University Hospital, and informed consent was obtained from each patient prior to initiating the study.

### Discussion

The following two distinct types of pineal-region meningioma have been reported: falcotentorial meningioma, and velum interpositum meningioma [1–3, 5, 6]. Meningiomas arising from the falcotentorial junction occur at the junction of the dural folds of the tentorium and falx cerebri and grow to occupy the quadrigeminal cistern by protruding anteriorly or inferiorly [1]. These tumors are usually fed by the tentorial branch of the meningohypophyseal trunk and occasionally by the medial and posterior choroidal arteries and branches of the posterior cerebral artery [5]. On the other hand, velum interpositum meningiomas are extremely rare and have been described as meningiomas in the posterior portion of the third ventricle that arise from the arachnoid membrane over the velum interpositum [4, 5, 7, 8, 10]. This type of tumor is fed by posterior choroidal arteries, and the ICVs are characteristically displaced upward [5]. No differences in clinical symptoms are seen among these two types of meningioma. Interestingly, Parinaud’s sign has been reported to be generally uncommon in patients with pineal-region meningiomas [3, 5, 11]. Indeed, neither of the present cases showed upper-gaze palsy. MRI and angiography are critical for correct diagnosis and pertinent surgical treatment for these meningiomas. In the present cases, angiography revealed that neither tumor received blood supply from either the tentorial artery or the choroidal arteries, and no upward displacement of the ICVs was present. These findings suggested that these tumors might not have originated from dural arachnoid cells of the falx or tentorium, and also not from arachnoid cells of the velum interpositum. Intraoperative findings demonstrated that the tumors did not entirely adhere to meningeal tissues of the falcotentorial junction or free edge of the tentorium, except for the surface of the vein of Galen.

Although thick and cloudy arachnoid membranes composing the posterior wall of the quadrigeminal cistern often hinder surgeons from approaching pineal tumors using the OTA, no detailed analysis of the microsurgical anatomy of the arachnoid membranes in the pineal region has been reported. One reason may be that most tumors of the pineal regions are malignant tumors such as germ cell tumors; which are invasive, and adhesion to the arachnoid is too firm to allow dissection for anatomical studies. In recent years, the distribution of the arachnoid membrane in the pineal region has been intensively examined. Vinas et al. provided the most detailed information about the AEPR [12]. Song-tao et al. described the supra- and infra-tentorial outer arachnoid membranes converging at the tentorial apex and then embracing forward along the vein of Galen to form the AEPR, which could be divided into the following two parts: the anterior AEPR (aAEPR), and posterior AEPR (pAEPR) [9]. Typically, the aAEPR envelops the suprapineal recess, pineal gland, and distal segment of the ICVs, and the pAEPR envelops the vein of Galen and terminal ends of its tributaries. We analyzed the anatomical features of the arachnoid membranes and deep veins, including the vein of Galen intraoperatively in the present cases. The tumor in Case 1 was tightly adhered to the inferior surface of the vein of Galen with thickened arachnoid membrane and displaced the splenium of the corpus callosum superiorly. The arachnoid membrane of this area became very thick and was stuck to the wall of the vein of Galen. Tumor adhesion to the thick membrane made dissection from the vein of Galen difficult. This tumor was presumed to originate from the inferior part of the arachnoid membrane over the vein of Galen. On the other hand, the tumor in Case 2 was attached to the superior surface of the vein of Galen and compressed the vein downward, with the splenium displaced anteriorly. The arachnoid in this region was easily dissected from the wall of the vein of Galen, resulting in complete resection of the tumor. This tumor was thought to have derived from the superior part of the arachnoid membrane over the vein of Galen. Such microsurgical studies of the arachnoid structures around the vein of Galen can be helpful to resect tumors more safely and to determine how completely a tumor should be resected.

The anatomical analyses of these cases may explain why the OTA is usually selected for tumors originating from the falcotentorial junction above the vein of Galen [1, 5, 13–15]. The approach side of the OTA might be decided based on consideration of the direction of tumor extension and the extent of development of collateral venous channels. On the other hand, the infra-tentorial supracerebellar approach is favored for some velum interpositum meningiomas that arise from the ventral half of the tela choroidea and displace the ICVs superiorly [4, 5]. In the present cases, both tumors occupied the quadrigeminal cistern within the level of the quadrigeminal plate, with anterior or superior deviation of the splenium of the corpus callosum. In addition, collateral venous channels had developed on the right medial surface of the occipital lobe in Case 1, and the tumor had extended to the right side in Case 2. Based on these findings, we selected OTA via the left side for resecting both tumors, and gross total removal of the tumors was achieved without any complications.

## Conclusions

We have reported two uncommon cases of pineal-region meningioma in which the origin was thought to be arachnoid cells in the arachnoid membrane over the vein of Galen. We demonstrated that the arachnoid membrane over the vein of Galen has a different nature at the upper and lower sides. Compared with the tumor at the inferior surface of the vein of Galen, adhesion of tumor to the superior surface of the vein of Galen was looser, and the tumor was more easily dissected from the vein. Among pineal-region meningiomas, we reported that tumors can derive from the arachnoid membrane of the vein of Galen and that these tumors are completely resectable by understanding the anatomy and nature of the arachnoid membranes in the quadrigeminal cistern.

## Consent

Written informed consent was obtained from the patients for publication of this case report and any accompanying images. A copy of the written consent is available for review by the Editor-in-Chief of this journal.

## Abbreviations

aAEPR: anterior arachnoid envelope over the pineal region
AEPR: arachnoid envelope over the pineal region
AMG: arachnoid membrane over the vein of Galen
CT: computed tomography
Gd: gadolinium
ICAG: internal cerebral angiography
ICV: internal cerebral vein
MRI: magnetic resonance imaging
OTA: occipital transtentorial approach
pAEPR: posterior arachnoid envelope over the pineal region
